# Homeostatic Proliferation and IL-7R Alpha Expression Do Not Correlate with Enhanced T Cell Proliferation and Protection in Chronic Mouse Malaria

**DOI:** 10.1371/journal.pone.0026686

**Published:** 2011-10-21

**Authors:** Robin Stephens, Benedict Seddon, Jean Langhorne

**Affiliations:** 1 Division of Parasitology, MRC National Institute for Medical Research, The Ridgeway, Mill Hill, London, United Kingdom; 2 Division of Immune Cell Biology, MRC National Institute for Medical Research, The Ridgeway, Mill Hill, London, United Kingdom; Université Pierre et Marie Curie, France

## Abstract

While chronic infection has been shown to enhance protection from disease caused by several pathogens, the mechanisms are not known. The gamma-c family of cytokines IL-7, IL-2, and IL-15 are implicated in homeostatic proliferation, which is thought to maintain T cell memory. However in chronic infection, prolonged antigen exposure itself may contribute to lymphocyte survival. We have previously observed that chronic malaria infection enhances protection to re-infection, as well as enhancing B cell responses. Here, we show that chronic *Plasmodium chabaudi* malaria infection in mice enhances the expansion of CD4^+^ T cells in a second infection, and that this correlates with increased expression of the IL-2/15 Receptor beta (CD122) on memory T cells, as well as increasing IL-2 producers on re-infection. IL-2 has been recently linked to improved secondary proliferation, while the role of IL-7 in maintenance of CD4^+^ memory cells has been demonstrated in homeostatic proliferation, but its role in protective memory populations in infectious disease protective has not been fully investigated. Increased IL-7Rα (CD127) expression correlated, as previously reported with increased turnover of CD4 memory cells, however, this was not linked to protection or enhanced response to rechallenge, These data support the idea that antigen or IL-2 production resulting from chronic stimulation may play a role in an enhanced secondary T cell response.

## Introduction

The common cytokine receptor gamma-chain family member IL-7 has been shown to be important for T cell development, survival and homeostatic proliferation for both CD4^+^ and CD8^+^ T cells[1], [2], [3], [4]. IL-7-deficient mice have greatly reduced numbers of T cells [5], [6] due to a combination of reduced development from the pro-T cell stage in the thymus [7], as well as a defect in post-thymic survival. While there is general agreement that CD8^+^ memory T cells depend on IL-7 signals for their homeostatic proliferation and survival [3], [8], the requirement for these signals for CD4^+^ T cells is not so clear. Functional CD4^+^ memory T cells seem to require both MHCII and IL-7 signals [9], [10], [11], and potentially other signals as well [12], [13]. IL-2 has been shown to contribute to CD4^+^-T cell survival by both upregulation of the anti-apoptotic factor bcl2, the cell cycle promoter c-myc [14], and IL-7Rα expression [15]. IL-2 has been shown to be important for secondary proliferation of CD4^+^ cells [16]. While studies of survival of antigen-specific CD4^+^ memory T cells have found a role for IL-7, other studies looking at the contribution of IL-7-dependent CD4^+^ Memory T cells to protection from Listeria and LCMV did not demonstrate a role for this cytokine [12], [13].

The role of persisting antigen on the functional capacity of CD4^+^ memory T cells is equally unclear. There is good evidence that CD4^+^ effector T cells can become memory cells in the absence of further TCR stimulation from MHCII [17]; however optimal memory cell function (enhanced sensitivity to low doses of peptide and stimulation by naïve B cells) may depend on a low affinity interaction with MHCII [18]. Furthermore, chronic infection and long-term antigen presentation has been described as important for protection against Influenza [19].

“Premunition” or resistance to reinfection in the presence of an existing infection is a feature of human malaria [20] and other chronic infections [21], and this supports the view that antigen in the form of chronic infection may be important in maintaining protective immunity. Indeed, in the mouse model of a blood-stage infection *Plasmodium chabaudi,* elimination of the chronic phase of infection with an antimalarial drug, chloroquine, results in higher parasitemias upon re-challenge with the homologous parasite [22]. Resistance to re-infection is dependent on CD4^+^ T cells via both antibody-dependent and independent mechanisms [23], [24]. However, the mechanisms of maintenance of memory CD4^+^ T cells in chronic infection are not known.

Here, we have examined survival and re-activation of CD4^+^ T cells in this *P. chabaudi* infection, and found that more CD4^+^ cells are activated on re-infection during chronic infection than when infection is eliminated after one month. This enhanced reactivation correlates with increased IL-2R beta expression on memory T cells and IL-2 in the second infection but not with IL-7R alpha expression or increased homeostatic proliferation in the memory phase of the response.

## Results and Discussion

### T cell Expansion in second infection is enhanced during chronic infection

**Figure 1:**
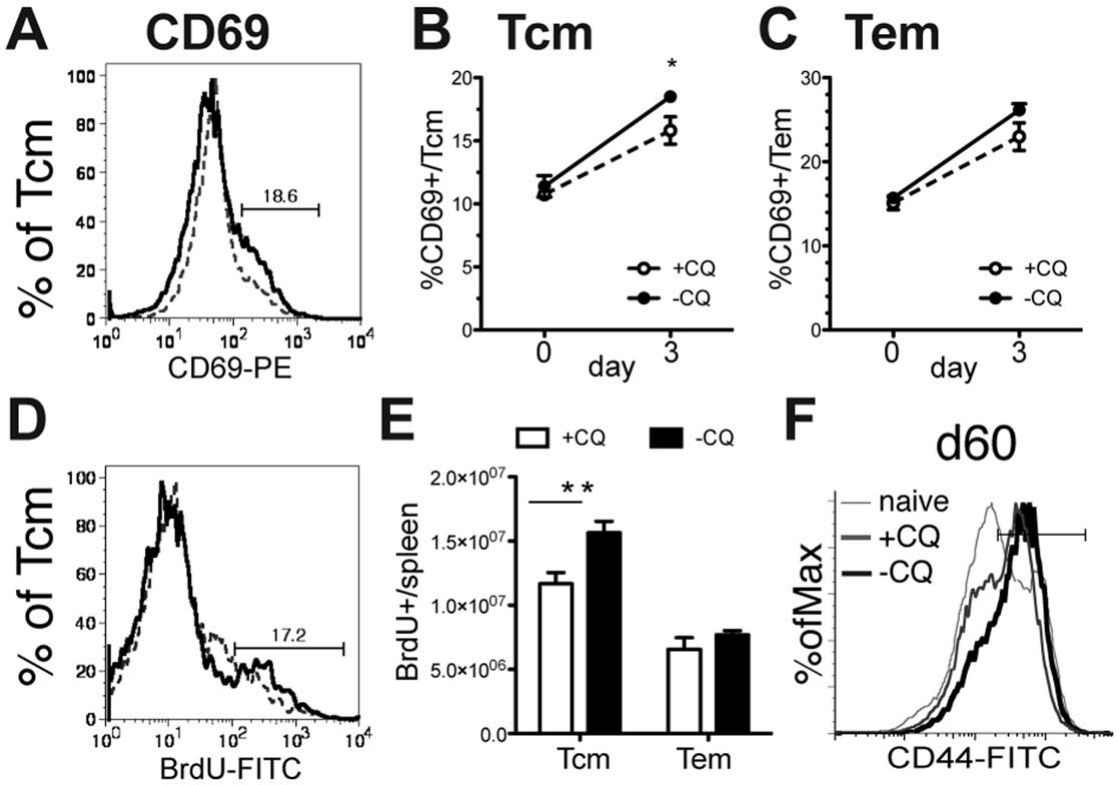
Chronic phase of a *P. chabaudi* infection enhances CD4^+^ Memory T cell activation and expansion. Mice were infected with 10^5^ *P. chabaudi* (AS). On days 30-34 one group of mice was treated with chloroquine (**+CQ**), which quickly eliminated the infection, while the other mice retained a chronic infection (**-CQ**). **A)** Half of each group was re-infected with 10^5^ *P. chabaudi* day 60 post-infection, and splenocytes were analyzed by flow cytometry on day 63 for CD4, CD44, CD62L and expression of the early activation marker CD69 (**A–C**) or incorporation of BrdU dosed into the water days 60–65 as an indicator of homeostatic proliferation (**D, E**), CD69 expression on central memory T cells (Tcm, CD44^hi^CD62^lo^) and effector memory T cells (Tem, CD44^hi^CD62L^int/hi^) are shown. Dotted lines represent chloroquine treated mice (+CQ) while bold lines represent chronic infection (-CQ). Data shown is the average of 4–5 mice per group and experiment was repeated twice with similar results. * indicates p≤0.05, ** p≤0.01.

A *Plasmodium chabaudi* blood-stage *infection* in C57Bl/6 mice becomes chronic for up to three months [22]. A second infection during the chronic phase leads to a reduced peak parasitemia compared with mice that have either cleared their infection naturally or been treated with anti-malarial drugs [22]. The mechanism of this improved protection is not known. In order to determine whether the extra protection afforded by chronic infection was accompanied by an enhanced CD4^+^ memory response, we analyzed their activation and proliferation in a second infection, comparing them with CD4^+^ memory T cells obtained from mice from which the chronic infection had been eliminated (**Figure 1**). C57Bl/6 mice were infected with 10^5^ *P. chabaudi*-infected red blood cells and then, after one month, some were treated with the anti-malarial drug, chloroquine (CQ) to cure the infection, preventing the chronic phase. The mice were re-infected, and activation of splenic CD4^+^ memory T cells by the second infection was measured as an increase in surface expression of the early activation marker CD69, on day 3, when its transient expression can be detected (**Figure 1A, B**), on both central (Tcm, CD44^hi^CD62L^hi^, **Figure 1A, 1B**) and effector/effector memory (Tem, CD44^hi^CD62L^lo^, **Figure 1C**) cells. Expression of CD62L was included in this analysis to allow discrimination between proliferation of central memory CD4+ T cells (CD62L^high^) and effector/effector memory CD4 T cells (CD62L^low^). In addition, the proliferation of T cells on re-infection was determined by measuring the incorporation of the thymidine analog, Bromodeoxy Uridine (BrdU) into dividing cells over the first five days of the second infection (**Figure 1D, E**), when they reach maximal numbers [24] and data not shown). Differences between chronic and treated mice are most clearly seen at day 60 and neither proliferation nor cytokines were detected well at day 3 (data not shown). As well as a significant increase in the proportion of activated Tcm in chronic infection seen in **Figure 1B**, the number of divided Tcm from mice reinfected during chronic infection (-CQ) was also greater (**Figure 1D, E**) than that of Tcm from mice treated with Chloroquine (+CQ) after 30 days of infection. While the trend was the same for Tem, only Tcm from chronically infected mice showed significantly enhanced activation. Interestingly, the proportion of CD44^hi^ CD4^+^ effector and memory T cells is enhanced in chronically infected animals before re-infection (**Figure 1F**). It is notable that the increases in activation and proliferation measured (using CD69 and BrdU) here reflect the combination of the effects of enhanced specific memory T cell frequency after clonal expansion as well as the enhanced intrinsic responsiveness of individual specific memory T cells to re-infection. There may also be an effect of non-specific stimuli resulting from the continued infection. While there is clearly an advantage to having more memory T cells in malaria [25], we have also shown that similar numbers of MSP1-specific memory cells from chronically infected animals protects RAG^o^ mice better than memory T cells from treated animals [**26**], suggesting that chronically stimulated memory cells are intrinsically more protective and that multiple effects are involved in the memory T cell response to malaria infection.

### IL-2 expression by chronically stimulated memory cells correlates with enhanced proliferation

**Figure 2:**
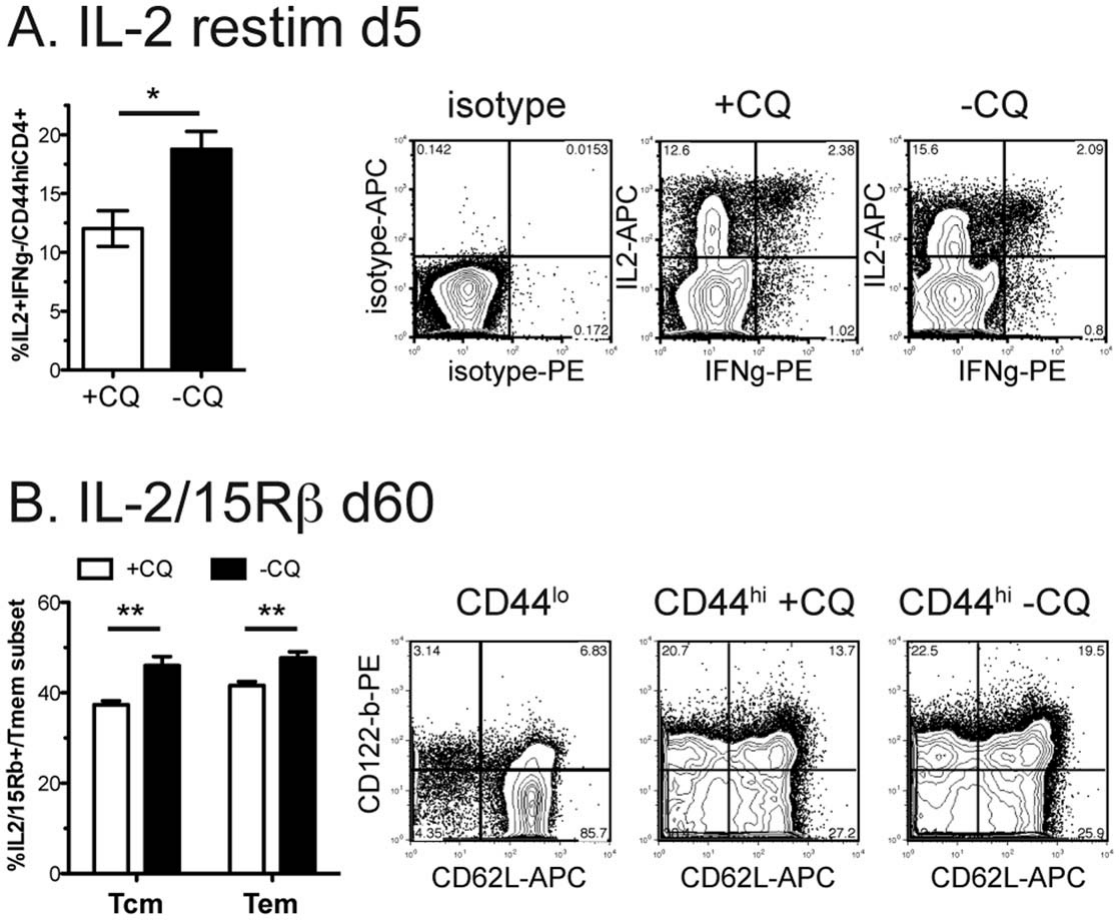
Chronic infection increases IL-2+ memory cells and IL2/15Rβ+ memory fraction. Mice were infected with 10^5^ *P. chabaudi*. On days 30–34 half of the mice were treated with chloroquine (**+CQ**), while the other mice retained a chronic infection (**−CQ**). Two months after infection, **A)** mice were re-infected and intracellular cytokine staining performed on day 5 of reinfection. Isotype control is shown to the left. **B)** Splenocytes were analyzed by flow cytometry for CD4, CD44, CD62L and IL-2/15R beta (CD122). Naïve cells (CD44^lo^) were used as an internal control to set the quadrants (left). Data shown is the average of 4-5 mice per group and experiment was repeated twice with similar results. Contour plots (10% with outliers) are gated as described on each plot and are from representative animals. * indicates p≤0.05, ** p≤0.01.

IL-2 is a growth and survival factor for activated T cells, and promotes their proliferation [7], [14], [16]. It is induced by stimulation of the T cell receptor (TCR). In our experiments, spleens of mice with chronic *P. chabaudi* infection (-CQ) and hence exposed to continuing antigen, contained a greater proportion of IL-2-producing memory T cells (IL2^+^IFNγ^γ ~^CD4^+^CD44^hi^) 5 days after re-infection (**Figure 2A**), compared with cells from infected mice treated with Chloroquine (+CQ), as measured by intracellular cytokine staining, which involves restimulation *ex vivo*. IL-2^+^IFNγ^−^ is the largest population, and may help the other cells survive, while IFNγ^+^ cells are likely to be effector and effector memory cells. A significantly greater proportion of these memory cells also expressed the beta subunit of the IL-2 and IL-15 receptors, CD122, (IL-2/15Rβ) (**Figure 2B**), supporting the hypothesis that chronically stimulated CD4^+^ memory cells can maintain themselves by autocrine IL-2. There is also some evidence that CD4^+^ memory T cells depend on IL-15 [27], a family-member of IL-2. CD122 has also been shown to be expressed on CD8^+^ memory T cells that specifically do not depend on MHC or antigen for survival and can use IL-15 for survival in homeostasis [28].

### Chronic infection reduces antigen-independent memory

**Figure 3:**
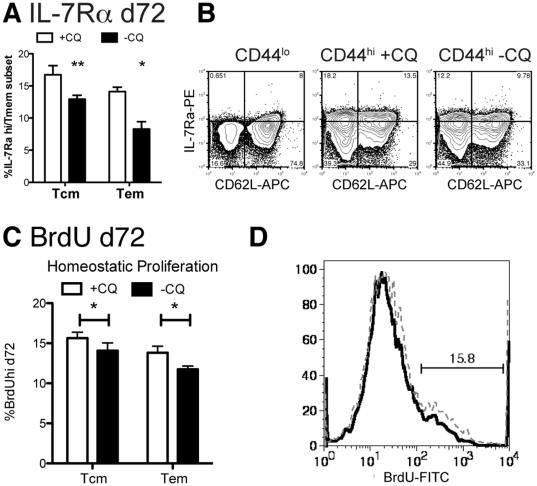
Chronic infection reduces IL-7Rα^hi^ memory cells in both memory subsets. Mice were infected with 10^5^ *P. chabaudi*. Days 30–34 half of the mice were treated with chloroquine (**+CQ**), while the other mice retained a chronic infection (**-CQ**). 2.5 months after infection, splenocytes were analyzed by flow cytometry for CD4, CD44, CD62L and (**A, B**) IL-7Rα (CD127) or incorporation of BrdU dosed into the water days 62–72 as an indicator of homeostatic proliferation, (**C, D**). Naïve cells (CD44^lo^) were used as an internal control to set the quadrants (**B**, left). Dotted lines represent chloroquine treated mice (+CQ) while bold lines represent chronic infection (-CQ). Data shown is the average of 4–5 mice per group and experiment was repeated twice with similar results. Contour plots (10% with outliers) are gated as described on each plot and are from representative animals. * indicates p≤0.05, ** p≤0.01.

IL-7 has been shown to promote survival of both naïve and memory CD4^+^ T cells and IL-7Rα has been shown in several studies to be up-regulated on antigen-independent memory T cells generated in acute infection [28], [29]. In order to investigate the use of this cytokine by CD4^+^ memory T cells in chronic infection, we measured the proportion of IL-7Rα (CD127)^hi^ CD4^+^ memory cells in this *P. chabaudi* infection. Naïve (CD44^lo^) splenic CD4^+^ T cells were IL-7Rα^lo^ [10] (**Figure 3**, left contour plot), allowing us to set the gate for this cytokine receptor, which has a slightly higher expression level on memory T cells (Tcm: CD44^hi^, CD62L^hi^; Tem: CD44^hi^CD62L^lo^, **Figure 3**, middle and right contour plots). In order to measure proliferation of T cells in the memory phase, BrdU was administered in the drinking water from days 62–72 post-infection. Interestingly, proliferation in this period was decreased in animals with chronic infection. This suggests that although they are better protected from re-infection [22], T cells do not have increased homeostatic proliferation.

A significantly greater proportion of CD4 T cells in both the Tcm, (CD62L^hi^) and Tem (CD62L^lo^) populations in chloroquine-treated mice express high levels of IL-7Rα, suggestive of their ability to use this cytokine for antigen-independent survival. These reciprocal changes of smaller proportions of IL-7Rαα and greater proportions of IL-2/15Rβ CD4^+^ memory T cells in chronic *P chabaudi* infection are indicative that both antigen-dependent and antigen-independent central and effector [26] memory cells are maintained. It is tempting to speculate that these cells can change their requirement for survival signals depending on the available antigen, as has been suggested for T cells in lymphopenic environments with high levels of IL-7 [27] and that IL-7 dependent and independent CD4+ memory cells may both play a role in protective immunity to reinfection.

Homeostasis has been reported to be a property of memory T cells that is required for their survival [28]. It has been suggested that although IL-7 enhances homeostatic proliferation [2], [11], and can help to determine the transition from *in vitro* effector T cells to *in vivo* memory T cells [10], as well as enhance CD4^+^ memory T cell survival [9], [12], IL-7 may not be essential for maintaining memory T cell [13]. IL-15 has been shown to be important in maintenance of CD4 T cell numbers in infection, but its role in protective memory has not yet been demonstrated [23]. Our data suggest that IL-2 [15], [16] and IL-15 [4], [27] may both play significant roles in survival, and re-activation of memory T cell, especially in chronic *P. chabaudi* malaria where the presence of antigen continually induces IL-2 and where more cells express IL-2/15Rβ (Figure 1A). Antigen duration during stimulation has also been shown to be important for the strength of the CD4^+^ proliferative response [30]. The expression of IL-7R alpha on T cells may also vary depending on the antigen, as it does at the peak where it marks CD8 cells to survive in LCMV [29], but not in peptide immunization [31]. Interestingly, autocrine IL-2 production by memory T cells on secondary stimulation has been shown to be important for proliferation to antigen by memory T cells [16], as we see in this chronic infection. These data therefore suggest that the maintenance or generation of IL-2 producing T cells by chronic infection may play a significant role in the enhanced proliferation seen in re-infection while high levels of IL-7R alpha and homeostatic proliferation may not indicate protective memory in chronic infection.

## Materials and Methods

### Ethics Statement

All experiments were approved by the ethical review panel at the National Institute for Medical Research and conducted under British Home Office regulations (PPL 80/2358).

### Mice and parasites

C57Bl/6 were bred in the National Institute for Medical Research under SPF conditions and for experiments maintained conventionally with sterile food and irradiated water *ad libitum*. Female 5–8-week-old mice were infected with 10^5^ parasitized erythrocytes from *P. chabaudi chabaudi* (AS) infected mice, and monitored by examination of Giemsa-stained blood films as described previously [32]. Chronic infection was eliminated by three i.p. injections of chloroquine (Sigma, UK) 50 mg/kg body weight in 0.9% saline solution (Sigma) at 2-day intervals, from days 30–34 of infection. This clone of *P. chabaudi* is sensitive to chloroquine when used at low parasite density [33]; after treatment no parasites were detectable by thin or thick blood film analysis or after sub-inoculation of blood into naïve recipients [22]. Primary and secondary infections were conducted simultaneously with age-matched uninfected controls and oldest uninfected mice are shown as day zero.

### Antibodies and flow cytometric analysis

Spleens were collected and dissociated into single cells in HBSS (Gibco, UK) containing 5% FBS (Seralabs, UK) and 6 mM HEPES. Erythrocytes were lysed using hypotonic lysis solution (Sigma). Nucleated cells were counted (Scharfe System CASY1, Reutlingen, Germany). Subset numbers were calculated by multiplying the percentage of lymphocytes by the total number of cells. Cells were stained at 3×10^6^/well in 96-well V-bottom plates and incubated with anti-CD16/32 (2.4G2) at 4°C for 20 min to block Fc binding. After washing, cells were incubated in PBS with 2% FCS and 0.1% Sodium azide and indicated combinations of FITC-, PE-, PerCP, TriColor-, biotin- or allophycocyanin- (APC)-conjugated antibodies with Strepdavidin -FITC or -APC (BD Biosciences, Cambridge Biosciences Oxford, UK). After washing, cells were fixed overnight with 2% paraformaldehyde (Sigma) in PBS. For intracellular staining, cells were stimulated for 5 hours with phorbol myristate acetate (PMA; 50 ng/mL;Sigma, UK), ionomycin (500 ng/mL; Sigma), and brefeldin A(10 g/mL; Sigma) for the last two hours. After surface staining, cells were fixed with Cytofix/Cytoperm solution (BDbiosciences). Fixed cells were permeabilized by washing in Perm/Wash buffer (BDbiosciences) twice and 20-min incubation. Cells were washed thrice in Perm/Wash buffer and re-suspended in staining buffer. A total of 30,000 lymphocytes were collected. Data acquired on a FACScalibur using Cell Quest Pro (Becton Dickenson) and analysed using FlowJo (Treestar, Portland, OR).

### Statistics

All experiments were analyzed by one-way ANOVAs and where differences were real, individual groups were studied by unpaired *t*-tests and *p*≤0.05 considered significant. Parasitemia was analyzed by the Mann Whitney non-parametric test. (Prism, GraphPad San Diego, CA).
